# Some Remarks on the Therapeutic Effects of Gossypium as an Emmenagogue and Parturifacient

**Published:** 1866-10

**Authors:** W. C. Bellamy

**Affiliations:** Columbus, Georgia


ATLANTA

### Medical and Surgical Journal.

NEW SERIES.

Vol. VIIB | OCTOBER, 1866. | No. 8.

### ORIGINAL COMMUNICATIONS.

### ARTICLE I.

*Some Remarks on the Therapeutic Effects of Gossypium as an Emmenagogue and Parturifacient.* By W. C. Bellamy, M. D., Columbus, Georgia.

Having been, during the last few years, on account of the late terrible and disastrous war, cut off from the rest of the world, by the blockade of our ports, and commercial intercourse with the northern part of our own country being closed, it became a matter of necessity with us to develop and apply to the alleviation of disease whatever indigenous remedies we might possess. Under the pressure of this necessity, many of our ablest physicians made numerous experiments in the practical use, not only of those substances which, though possessing medical properties, had never been used to any appreciable extent, but also to bring to light the medical properties of many articles hitherto unknown to the profession.

Being myself, among the rest, subjected to the same inconvenience, necessity compelled me to experiment with and finally to develop, to my entire satisfaction, the therapeutic effects of the gossypium, (our southern staple product, the common cotton plant,) as an emmenagogue and parturient. Needing, but not being able to obtain, the ergot, and remembering the observations of Dr. Bouchelle, of Mississippi, in regard to this plant, as recorded on page 388 of the U. S. Dispensary, edition of 1858, I determined to put it to the test. The paragraph referred to says: "The root of the plant has been employed by him, and he believes it to be an excellent emmenagogue, and not inferior to ergot in producing uterine contractions. He also states, that it is habitually resorted to by the slaves of the South for producing abortion; and he believes it acts in this way without injury to the general health." To assist labor, he makes a decoction by boiling four ounces of the root in a quart of water to a pint, and gives a wine-glassful every twenty or thirty minutes.

The first case in which I tried the remedy was a negro woman, patient of one of my confreres. It was a case of tedious labor, and not being able to obtain the ergot, a consultation determined him to try my new remedy. But the large size of the dose, and the shortness of the intervals, given as recommended by Dr. Bouchelle, deterred me, fearing the bulk would overload the stomach, and nauseate the patient too much. I therefore prepared a compound fluid extract, and concentrated it, so as to reduce the size of the dose to a tea-spoonful, gave him a bottle of it with directions to give a tea-spoonful every twenty or thirty minutes, requesting him to give me the result of his observations. On the following morning, he called upon me and informed me that the medicine had subjected him to a considerable degree of mortification; that the labor was progressing very slowly; that he had little confidence in the medicine, and that he thought he would have ample time to visit another patient near by. Gave a dose of the medicine, and leaving the bottle with directions to give a tea-spoonful every twenty minutes, went to visit another patient; was gone perhaps three-quarters of an hour, or an hour at farthest, and returned to find to his no less astonishment than mortification, that the labor was completed—that the woman had given birth to a good-sized, healthy child, with very little trouble, after having taken not more than two or three doses of the extract of gossypium.

Soon afterwards I had occasion to use it myself in several cases of tedious and protracted labor (no one case where the labor had lasted over forty-eight hours) with the same happy results, and I had the pleasure of seeing all the patients, to whom I had given it, go into a rapid state of convalescence, without observing a single pernicious symptom in any of the cases. Finding the remedy to act so charmingly as a parturient, I felt sure it would succeed equally in amenorrhœa and dysmenorrhœa. I, fortunately, soon had an opportunity of trying it in the former, as well also as in dysmenorrhœa. The patient was Mrs. W., a most estimable lady of full plethoric habit, short neck, weighing about two hundred pounds, and about 35 years of age. She had been troubled with a suppression for more than twelve months, and had been at irregular intervals troubled in the same way for some years previously. She was troubled with it at this time, as I said, for more than twelve months, with all the accompanying unpleasant symptoms, when she applied to me. After the exhibition of aloetic laxatives, I gave her my new remedy, with which she was to commence about a week before her regular period and take a tea-spoonful night and morning until the day arrived for the regular menstrual discharge, when she was ordered to take her bed, make her feet and body warm between blankets, and take a tea-spoonful of the extract of gossypium every hour until some show should appear, when, towards evening, to her great relief and delight, she discovered a little show. But that period passed by without much benefit. She was, however, ordered to repeat the same treatment at her next period, which she did, and was entirely restored, having used an aloetic laxative occasionally, as she was continually constipated.

Shortly after this, I had another opportunity of trying it in a case of dysmenorrhœa, in a robust, sanguine young widow. To her I gave during the period a tea-spoonful every half hour, under which treatment the discharge soon became natural and without any pain.

I am fully satisfied, from the experiments and impartial trials I have given the remedy, that it is fully equal, if not superior, to ergot in promoting the various functions of the uterine organs.

I look upon it as a sure, speedy and safe remedy, not only for difficult, painful, contracted labors, but also to control all the irregularities of females and to alleviate their peculiar monthly sufferings. It is very certain that its effects are so powerful upon the uterine system as to produce miscarriage, if administered during pregnancy. I feel that its merits cannot be too highly extolled, and hope you will call attention to it and that it may be brought into general use. It is too valuable a remedy to remain hidden in the depths of obscurity. I have made arrangements to have it prepared from the recipe I used after the manner of Tilden's fluid extracts, for the convenience of physicians who may like to use it. I consider it preferable to ergot.

The proper time to gather the root is, when it is as old as possible without being injured by the severe frosts; therefore it is best when gathered during the months of October and November. If gathered before October, it is not sufficiently matured to possess its virtues to the fullest extent, and if taken later than November, it is apt to be injured by the frost. As soon as I am able to get some of the extract prepared, I will send you a bottle of it with the desire that you try it and give us, through the medium of your valuable Journal, the result of your experiments and observations on its use. I do not propose to prepare it as a patent medicine, for it is too great a blessing to suffering females not to place it within the reach of all.

